# Neuroimmunologic and Neurotrophic Interactions in Autism Spectrum Disorders: Relationship to Neuroinflammation

**DOI:** 10.1007/s12017-018-8488-8

**Published:** 2018-04-24

**Authors:** Kshama Ohja, Evelyne Gozal, Margaret Fahnestock, Lu Cai, Jun Cai, Jonathan H. Freedman, Andy Switala, Ayman El-Baz, Gregory Neal Barnes

**Affiliations:** 1 Department of Neurology, University of Louisville School of Medicine, Louisville, KY USA; 2 Department of Pediatrics, University of Louisville School of Medicine, Louisville, KY USA; 3 Department of Psychiatry and Behavioural Neurosciences, McMaster University, Hamilton, ON Canada; 4 Department of Pharmacology and Toxicology, University of Louisville School of Medicine, Louisville, KY USA; 5 Department of Bioengineering, University of Louisville, Louisville, KY USA; 6 Spafford Ackerly Chair in Child and Adolescent Psychiatry, University of Louisville Autism Center, 1405 East Burnett Avenue, Louisville, KY 40217 USA

**Keywords:** Brain-derived neurotrophic factor, ASD, T cells, Cytokines, Microglia, PI3 kinase signaling

## Abstract

Autism spectrum disorders (ASD) are the most prevalent set of pediatric neurobiological disorders. The etiology of ASD has both genetic and environmental components including possible dysfunction of the immune system. The relationship of the immune system to aberrant neural circuitry output in the form of altered behaviors and communication characterized by ASD is unknown. Dysregulation of neurotrophins such as BDNF and their signaling pathways have been implicated in ASD. While abnormal cortical formation and autistic behaviors in mouse models of immune activation have been described, no one theory has been described to link activation of the immune system to specific brain signaling pathways aberrant in ASD. In this paper we explore the relationship between neurotrophin signaling, the immune system and ASD. To this effect we hypothesize that an interplay of dysregulated immune system, synaptogenic growth factors and their signaling pathways contribute to the development of ASD phenotypes.

## Background

### Introduction

Autism spectrum disorder (ASD) is a developmental disorder characterized by limitations in social and behavioral characteristics including limited communication/social interactions, sensory abnormalities and repetitive interests and behavior. (Polleux and Lauder 2004; APA 2000). It has a prevalence ranging from 0.7 to 2.64% (Gesundheit et al. 2013). ASD is a widely used term encompassing a large spectrum of disorders previously recognized in the DSM-IV. Autistic disorder, Asperger Syndrome, Pervasive Developmental Disorder Not Otherwise Specified (or atypical autism), Childhood Disintegrative Disorder and Rett Syndrome (Gentile et al. 2013) were all individual disorders recognized under DSM-IV. The DSM-V criteria recognize just one disease, Autism Spectrum Disorder, which manifests before 3 years of age (Stromland et al. 1994; Rodier et al. 1996; Rodier 2000). ASD is associated with a number of co-morbidities including epilepsy, gastrointestinal dysfunction, psychiatric disorders, insomnia, intellectual disability and decreased motor tone/motor skills (Gesundheit et al. 2013).

### Proposed Pathogenesis

Both genetic and environmental factors contribute to ASD (Goines and Ashwood 2013). ASD pathogenesis comprises many disparate mechanisms including chronic neuroinflammation, GABAergic imbalance, monoaminergic dysregulation and mitochondrial dysregulation (Vargas et al. 2005; Morgan et al. 2010; Young et al. 2011). While these factors are proposed to play major roles in the pathogenesis of ASD, immune dysregulation, microglial activation and genetic contributors to immune dysregulation may be prime treatment targets in the pathogenesis of ASD (Careaga et al. 2010; Goines and Ashwood 2013; Ahmad et al. 2017a, b). Altered connectivity of neuronal networks is thought to underlie the clinical symptoms of ASD. Recent studies implicate molecules involved in synapse development and plasticity in the etiology of autism (Bourgeron 2015). Among these are brain-derived neurotrophic factor (BDNF), its receptor, TrkB and the proteins involved in their signaling pathways affecting dendritic spine protein synthesis and stability. The transforming growth factors (TGFβ 1, 2, 3) are crucial regulators of the immune system and cellular homeostasis and play a critical role in the regulation of inflammation (Aoki et al. 2005). Decreased TGFβ plasma levels correlating with worsening behavioral measures were reported in a cohort of ASD children (Ashwood et al. 2008). The goal of this review is to identify multiple factors and discuss their potential interactions that may lead to the development of neuroinflammation contributing to ASD symptoms. We hypothesize that the interplay of environmental and genetic factors results in dysregulated immune system via TGF-β signaling and impairs neurotrophin signaling pathways, contributing to the development of ASD phenotypes. Through TGF-β/Wnt signaling, these interactions result in alterations of synaptic function and circuitry which lead to the development of ASD phenotypes.

## Immune System and ASD

Our body’s defense system comprises both innate and adaptive immunity. An initial immune response is mounted by innate immunity, which recognizes pathogen-associated molecular patterns, also known as PAMPs (Medzhitov and Janeway 2000). PAMPs recognized by pattern recognition receptors activate their innate immunity cells to produce certain pro-inflammatory and counter-regulatory cytokines, which mediate phagocytosis of the pathogenic agent. Innate immunity paves the way for the development of adaptive immunity. CD4 + T cells, upon interacting with APCs, develop into Th1 and Th2 T cells. These T cells cross the blood brain barrier and secrete cytokines, dependent upon the type of target antigen as presented to them by the CNS APCs. Distinct T cell subtypes secrete different cytokines that counter-regulate each other. Evidence suggests that an imbalance between pro- and anti-inflammatory pathways plays an important role in the pathogenesis of several neurodevelopmental disorders including autism (Romagnani 1997; Swain 1999; Medzhitov and Janeway 2000; Wright 1999; Ahmad et al. 2017a, b; Flavell 1999).

The onset of autistic behavior or worsening of the same often occurs after common childhood illnesses. Children with ASD have adverse reactions to benign factors like immunizations, common illnesses and environmental challenges (Jyonouchi et al. 2014). Clinical reports suggest that aberrant behavior may improve in some febrile ASD children (Curran et al. 2007). A major co-morbidity of ASD is its GI manifestations such as diarrhea, constipation, gastroesophageal reflux and colic. These children present with lymphoid nodular hyperplasia and inflammation of the GI mucosa and with distinct innate immune abnormalities and peripheral blood monocyte alterations (Li et al. 2017; Jyonouchi et al. 2011). Immune dysregulation, with increased levels of pro-inflammatory cytokines produced by peripheral blood mononuclear cells, has been observed frequently in individuals with ASD (Ashwood et al. 2008; Jyonouchi et al. 2011; Bilbo et al. 2012; Persico et al. 2012; Enstrom et al. 2009; Jyonouchi et al. 2001; Molloy et al. 2006; Vargas et al. 2005; Zimmerman et al. 2005; Torres et al. 2012).

**Fig. 1:**
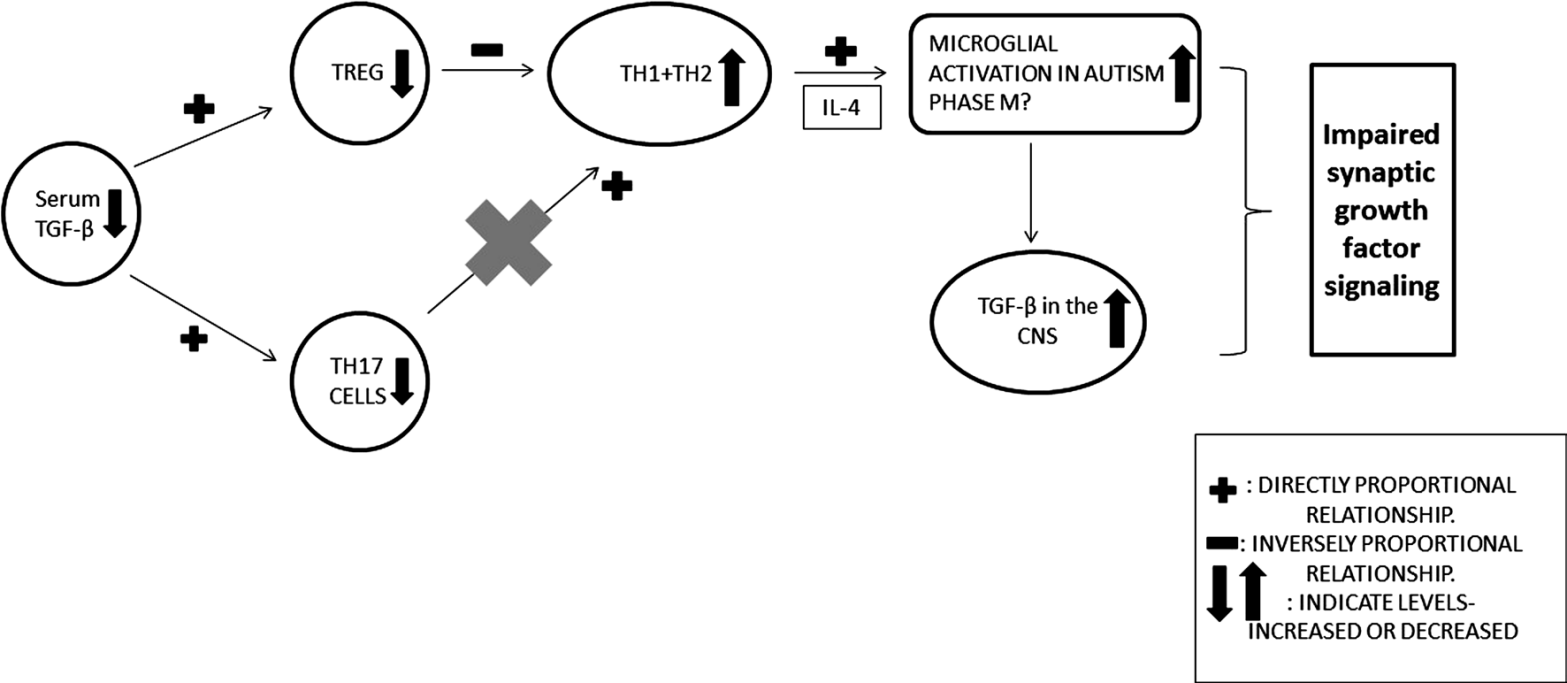
Effect of Decreased Peripheral TGF-β Signaling on T Cell Subtype Physiology in ASD. Decreased serum TGF-β leads to decreased production of Th17 and Treg cells. Loss of Treg cell regulatory control leads to increased Th1 and Th2 cell activity. With increased Th2 cell activity, increased interleukin production including IL-4 causes microglial activation of altered M (M2?) phenotypes. The activated microglia cell population increases a variety of components including increased CNS TGF-β

The roles of immune mediators, especially cytokines, overlap considerably in order to maintain homeostasis in the human body. However, pro-inflammatory and anti-inflammatory roles have been assigned to various cytokines through extensive studies (Dhabhar 2014). ASD commonly presents with an elevation of pro-inflammatory cytokines such as *IL***-***6* (Th2)*, TNF***-***α* (Th1), *GMCSF* and *IL***-***8* (multiple immune cells) and a decline in the level of anti-inflammatory cytokines (*TGF***-***β, IL***-***10*) in the blood (Fig. 1) (Ashwood et al. 2004, 2008; Croonenberghs et al. 2002; Li et al. 2009; Ashwood et al. 2011). Studies have also shown elevated levels of nuclear factor kappa-light-chain enhancer of activated B cell, also known as *NF***-***κB,* in blood and brain of ASD patients (Young et al. 2011). NF-κB is a transcription factor essential to the regulation of cytokines and of B and T cell receptor expression and which plays an important role in the development of the CNS (Memet 2006). Decreased levels of immune mediators such as TGF-β1 are associated with worsening symptoms among ASD afflicted children (Ashwood et al. 2008; Goines and Ashwood 2013). Several studies have also shown decreased lymphocyte numbers (Ashwood et al. 2003), skewed T helper cells’ cytokine profiles and altered T cell and monocyte responses (Molloy et al. 2006; Enstrom et al. 2010), all pointing toward immune dysregulation as depicted in Fig. 1. While the existence of immune dysregulation in ASD is clear, its pathology, pathogenesis and whether its pro- or anti-inflammatory effects underlie the disease remain to be understood.

## Hypothesis 1: Immune Dysregulation in ASD

Impaired cell signaling of the immune system via TGF-β and Th1/Th2/Th17/Treg interactions along with environmental factors contributes to the pathophysiology and treatment responses in ASD via synaptogenic growth factor(s)-mediated mechanisms (Fig. 1).

## Transforming Growth Factor Beta (TGF-β): Role in ASD and Development of T Helper Cell Subtypes

The role of transforming growth factor beta (TGF-β) in the development of different subtypes of immune T cells is an integral one, and any alteration in TGF-β levels can result in immune dysregulation (Mantel and Schmidt-Weber 2011). Vargas et al. (2005) found that TGF-β was one of the most prevalent cytokines in brain tissues of individuals with ASD. The levels of TGF-β in serum are inversely related to those in the brain of autistic individuals (Ashwood et al. 2004).

Immune processes, previously unexplained by Th1/Th2 actions/interactions, have undergone a revolution with the discovery of Th17 cells. Th17 and Treg cells are both subsets of T cells which derive from a common precursor cell but have opposing effects, i.e., pro-inflammatory and anti-inflammatory, respectively (Fig. 1; Mantel and Schmidt-Weber 2011). Th 17 cells are activated by IL-23 and TGF-β to produce IL-17. Studies have reported decreased levels of IL-23 and TGF-β in children with ASD (Ashwood et al. 2008; Yang et al. 2008). Other studies have also shown decreased levels of IL-23 but normal levels of IL-17 and a negative correlation between the levels of IL-23 and higher ADOS (Autism Diagnostic Observational Schedule) scores in children with ASD (Onore et al. 2009).

Studies on mouse models have shown that decreased numbers of T cells and their interactions with microglia could lead to impaired learning and memory via an effect on neurogenesis by the interaction between both T cells and microglia (Onore et al. 2009). On the other hand, the roles played by cytokines produced by Th17 cells are ambiguous in the case of ASD. For example, in the mouse model of maternal immune activation (MIA), increased IL-17 is required to produce the abnormal cortical phenotype and ASD-like behavior in offspring (Choi et al. 2016). The role of maternal brain autoantibodies, which could be linked to these cytokines, remains unclear in ASD but could be increased in inflammatory conditions during pregnancy (Krakowiak et al. 2017a, b). For a fuller discussion of maternal autoantibodies, the reader is referred to Edmiston et al. (2017). However, it is clear that the immune system must remain in balance, as either hyper- or hypo-activity of the immune system may result in abnormalities in behavior and cognition (Ziv et al. 2006; Ziv and Schwartz 2008).

While naturally occurring thymic Treg cells (nTregs) develop during thymic selection, independently of TGF-β, induced Treg (iTreg) cell production requires TGF-β (Lan et al. 2005). TGF-β has also been known to suppress Th1 and Th2 cell production. The interaction between T helper cells and their subtypes, especially Th1/Th2/Th17/Treg, exists in ASD, and recent studies have shown that children with ASD had decreased Foxp3 + Treg cells and increased transcription factors produced by CD4 + T cells (Ahmad et al. 2017a, b). These data further illustrate the important role of TGF-β in the production of all T helper cell subtypes and their potential contribution to brain pathophysiology (Fig. 1).

### Roles of Th1 and Th2 Cells in CNS Microglial Activation

Microglial cells are the innate immune cells of the CNS that act as antigen-presenting cells (APCs). Th1 and Th2 cells interact with microglia (Fig. 1) which express other co-stimulatory molecules, upon activation by inflammatory stimuli. Microglia also stimulate T cells to secrete immune mediators that serve as regulators of T cell phenotype, activation and recruitment within the CNS. Astrocytes may also play a role in the inflammation cascade. Astrocytic activation and stimulation potential is limited to the Th2 type of T cell in the presence of antigen proteins (Aloisi et al. 2000).

Microglial activation of T cells brings them into contact with specific antigens, which results in the production of various cytokines. Murphy et al. (2010) conducted a study demonstrating that in experimental autoimmune encephalitis (EAE), IL-17^+^ and IFN-γ^+^ IL-17^+^ T cells infiltrated the CNS prior to the onset of clinical symptoms. This phenomenon was associated with activation of microglial cells and increased expression of IL-1β and IL-6 (produced by microglia/macrophages) in the brain (Murphy et al. 2010; Sutton et al. 2006). Thus, microglial activation has been hypothesized to be linked with Th17 cells and their interactions, resulting in the production of significant levels of pro-inflammatory mediators as seen in EAE (Murphy et al. 2010). McQuillan et al. (2010) conducted another study that lends support to the hypothesis that Th17 activates microglia cells in the CNS. Their data suggested that Aβ-specific Th 1 and Th17 cells activate microglial cells in the CNS, whereas Th2 cells have a regulatory effect on these cells (McQuillan et al. 2010). While there is a considerable lack of evidence regarding the role of Treg cells in microglial activation in humans, studies in mice have provided significant evidence regarding these interactions (Xie et al. 2015), establishing the link between Th17, Treg cells and microglial activation (Fig. 1). The interaction between Th17/Treg/microglial activation has not been extensively studied in ASD. Thus, it becomes imperative to further study the association between immune dysregulation involving Th17 cells, Treg cells and microglial activation in ASD. Overall, these findings strongly suggest a role for the dysregulation of immune function and for TGF-β in the development of neuroinflammation and ASD symptoms. While T cell alterations may underlie changes in immune function, microglial recruitment and activation play a significant role in the onset of neuroinflammation.

## Microglial Cell Activation and ASD

Microglial cells belong to the mononuclear phagocyte series of cells, are exclusively present in the CNS and have a myeloid origin. A major migration of myeloid progenitor cells into the CNS takes place during development and results in microglial formation. Microglia in the CNS usually remain in an inactive state. Resting microglia contribute to immune homeostasis, clearing debris, secreting cytokines and growth factors and communicating with surrounding neurons. They are activated when exposed to triggers and can then carry out diverse functions which could prove to be either effective or harmful (Chan et al. 2007; Ransohoff and Perry 2009). The presence of microglial activation in ASD has been established by multiple studies including Vargas et al. (2005), who detected a marked increase in neuroglial response (activation of microglia and astroglia) in the brains of patients with ASD. Immunocytochemical analysis of neuroglial reactions in the brain demonstrated that regardless of differences in demography, past medical history or associated co-morbid conditions, morphological changes in the brain were consistent with persistent and chronic inflammatory processes in cortical and subcortical white matter and cerebellum. Increased production of both anti- and pro-inflammatory cytokines by neuroglial cells was observed. Western blot analysis also revealed increased levels of GFAP, an indicator of astroglial activation, in patients with ASD as compared to controls. The pattern of these inflammatory responses in ASD resembled those seen in neurodegenerative disorders such as Alzheimer’s disease, Parkinson’s disease and amyotrophic lateral sclerosis (Vargas et al. 2005).

Microglial cells produce pro- and anti-inflammatory cytokines in response to various pathologic stressors and, like macrophages, have been shown to have different phenotypes. Analysis of inflammatory markers expressed by microglia in their various phenotypes suggests that they are somehow similar to their respective macrophage phenotypes (Chhor et al. 2013; Franco and Fernandez-Surarez 2015). The M1 (Classical) phenotype in microglia appears in response to pro-inflammatory signals and is cytotoxic in nature (Ransohoff and Perry 2009; Chhor et al. 2013; Franco and Fernandez-Surarez 2015). On the other hand, M2 (Alternative) phenotypes of microglia are involved in repair and regeneration by producing anti-inflammatory cytokines and form the deactivating phenotype (Chhor et al. 2013; Franco and Fernandez-Surarez 2015). The role of cytokines in phenotype conversion remains the same for both microglia and macrophages. M1 activation, characterized by activation of ERK1/2 and p38, MHC-II expression, cytokine production (TNFα, IL-1β, IL6, IL12) and oxidative stress (iNOS, COX-2, ROS and glutamate), can potentially drive inflammatory and neurotoxic responses. M2 is an alternative activation state, induced by IL-4 and IL-13 and characterized by Ym1, FIZZ1 and Arginase 1 expression, which appears to be beneficial, i.e., involved in tissue repair and remodeling and removal of debris. M2 activation can be divided into M2a for repair and regeneration through anti-inflammatory and immune regulatory secretion, and M2b/c which presents a deactivating phenotype, expressing mainly anti-inflammatory markers (Mantovani et al. 2004).

In cultured microglia and macrophages, the M2 activation profile is initiated by IL-4, a cytokine secreted by Th2 cells, as well as by IL-13 (Mantovani et al. 2004). Multiple studies have revealed that patients with ASD have increased levels of IL-4 and of IL-4-producing Th2 cells as compared to controls (Fig. 1; Gupta et al. 1998). A study in neonates has shown that levels of cytokines at birth are associated with ASD later in life and that increased levels of IL-4 are associated with increased odds of having severe ASD in the future (Krakowiak et al. 2017a, b).

## Impact of Genetics on Immune Signaling in Autism

Genetic evidence for ASD is strongly supported by the 3–19% recurrence risk in families with a 60–90% overlap of the rate of diagnosis in monozygotic twins, and a 10–30% overlap in same-sex dizygotic twins (Bailey et al. 1995; Colvert et al. 2015; Frazier et al. 2014; Hallmayer et al. 2011). Only 10% of the cases in ASD are syndromic with the rest being non-syndromic or idiopathic with ambiguous origins (Persico and Bourgeron 2006; Lintas and Persico, 2009). Genome scans and sequencing studies have revealed 15–20 loci and hundreds of genes associated with non-syndromic cases whose interactions and exact roles are complicated by genetic heterogeneity, inter-genetic interactions, incomplete penetrance and phenocopies (Devlin and Scherer 2012; De Rubeis et al. 2014); Persico and Bourgeron 2006). Besides high penetrance monogenic disorders like Fragile X Syndrome (Berry-Kravis et al. 2017), prominent genes that have been identified include Reelin (RELN) (Quattrocchi et al. 2002), MET (Campbell et al. 2008), serotonin transporter (5HTT) and engrailed 2 (EN2) (Bartlett et al. 2005). In addition, genome-wide mRNA expression studies on brain tissue of patients with ASD revealed approximately 186 upregulated and 35 downregulated genes in comparison with controls (Garbett et al. 2008). 37.3% of the differentially expressed transcripts were either immune-related or cytokine-responsive transcripts (Garbett et al. 2008).

Several genes associated with ASD also have been genetically linked to autoimmunity and to immune-based genes like HLA-DRB1 and complement C4 (Table 1; Estes and McAllister 2015). Upregulated genes identified by genome-wide mRNA screening fell into 31 gene sets; approximately 19 of these sets were associated with immune signaling: targeting of the immune response to specific cells (NKT pathway), inflammation (NF-κB, IL1R, IL6, P38MAPK, INFLAM, GSK3 and TH1/TH2 pathway), apoptosis (NF-κB, TNFR2, P38MAPK, TID, 41BB, CASPASE and FAS pathway), autoimmune diseases (NF-κB, TOB1 and FAS pathway), migration (MCALPAIN pathway) and antigen-specific immune response (TOLL, TNFR2, HIVNEF, DC and IL2R pathway) (Garbett et al. 2008). Upregulation of these genes suggests dysregulation of the immune system in the brain of autistic patients affecting their ability to successfully downregulate activation of cytokines and microglia (Garbett et al. 2008). Thus, immunogenetic factors may likely contribute to ASD pathophysiology (Table 2).

**Table 1:** Autism-Associated Genes with Immune Functions

Gene	CNS function of encoded protein	Immune function of encoded protein
*JARID2*	Transcriptional repressor involved in neural tube fusion	Transcriptional repressor that regulates haematopoiesis
*TPO*	Necessary for proper brain development	Enzyme that produces thyroid hormones
*MET*	Mediates migration of neuronal precursors and excitatory synapse formation	promotes differentiation and Proliferation of haematopoietic cells and exerts broad anti-inflammatory effects
*PRKCB*	Implicated in circadian rhythms and learning and memory	Mediates B cell activation, T cell migration, antigen-presenting cell function and cytokine release
*HLA*-*A2*	Negatively regulates synapse formation and plasticity in the developing brain	Expressed on all nucleated cells to identify them as ‘self’ to immune cells; regulates cellular immune responses to intracellular pathogens
*HLA*-*DRB1*	Unknown	Initiates cellular immune responses to extracellular pathogens
*C4B*	May be involved in complement-mediated synaptic pruning	Complement cascade protein that is involved in clearing pathogens and cellular debris

**Table 2:** Microglial activation

Boche et al. (2013)	M1 (classic activation)	M2 (alternative activation: wound healing/regulatory)
Stimulus	Interferon-γ, TNF-α	IL-4, IL-13, TREM2
Source	Natural killer, T helper 1 lymphocytes	Macrophages, granulocytes responding to tissue injury, fungi and parasites (chitin), T helper 2 lymphocytes
Macrophage products	Pro-inflammatory cytokines: IL-1β, TNF-α, IL-6, IL-23 Oxygen free radicals	TGF-β, Arginase 1, Chitinase, Extracellular matrix components
Cell surface proteins	MHC-II	Mannose receptor (CD206)
Functions	Kill micro-organisms and other cellular targets. Phagocytosis Present antigen to lymphocytes. May cause collateral damage to host cells.	Tissue repair/wound healing. Phagocytosis. Increases production/remodeling of extracellular matrix. Inhibits inflammation

## Summary of Immune Signaling in ASD

Microglial cells are the resident immune cell of the CNS. They are normally present in the brain and can respond to injury by activation, resulting in phenotypic and morphological changes, chemokine secretion and attraction of blood-borne macrophages to the site of injury (reviewed in Franco and Fernandez-Surarez 2015). Similar to macrophages, microglia can undergo two distinct types of activation, M1 and M2, which might lead to different phenotypic characteristics and secretion profiles from those of macrophages. In ASD, decreased serum/blood TGF-β levels lead to decreased TREG and TH17 cells, which control TH1 and TH2 levels, thus leading to their increased numbers and cytokine (IL-4) levels (Fig. 1). According to the hypothesis 1, the influence of dysregulated T cell function further leads to increased microglial activation in the brain including increased M2 microglia (elevated IL-4 levels) which contributes to increased TGF-β levels in the ASD brain parenchyma, thus impairing synaptic growth factor signaling (Fig. 1).

## Hypothesis 2: Immune-Synaptogenic Growth Factor Interactions in ASD

**Fig. 2:**
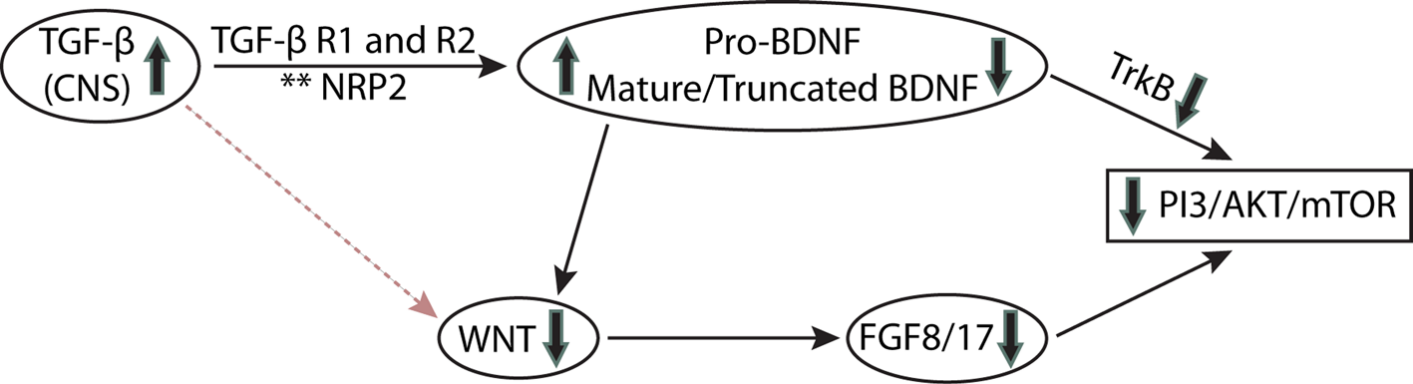
TGF-β influences on neurotrophic signaling in ASD. Increased TGF-β levels through its receptors [TGF-β R1/R2 and putative Neuropilin 2 receptors (Gant et al. 2009)] may cause altered BDNF/TrkB processing such that neurons produce increased pro-BDNF and less full-length TrkB (TrkB-FL). [Make it TrkB-FL in the figure.] Decreased TrkB signaling combines with decreased FGF8/17 signaling to produce additive decreases in PI3K/Akt/mTOR signaling. The decreased FGF signaling is likely due to the negative effect of increased TGF-β signaling on WNT signaling. **NRP2 = Neuropilin 2 which is a binding partner of TGF-β receptors

We hypothesize that immunogenetic-environment interactions through TGF-β/Wnt signaling alter neural circuitry development and thereby behavior in children and adolescents with ASD via synaptogenic growth factor(s)-mediated mechanism(s) (Fig. 2).

### BDNF and Autism

BDNF is a powerful neurotrophin/synaptogenic growth factor with various CNS functions relevant to ASD including neuronal differentiation, neuronal survival and migration, synaptogenesis, synaptic plasticity and facilitation of GABAergic neuronal phenotype development (Barde et al. 1982; Huang and Reichardt 2001; Lu et al. 2009). BDNF is among the most widely expressed neurotrophic factors in the post-natal brain, with significant levels in the neocortex and hippocampus (Wetmore et al. 1990).

Genetic studies provide direct evidence of the involvement of BDNF and its receptor, TrkB, in the pathogenesis of ASD. A specific BDNF SNP (single nucleotide polymorphism) and other BDNF haplotypes, linkage peaks and NTRK2 (TrkB) gene variations are associated with autism, bipolar disorder, schizophrenia, OCD and ADHD (Correia et al. 2010; Nishimura et al. 2007; Fanous et al. 2004; Alonso et al. 2008). Mice with Ntrk2 gene mutations showed reduced synaptic plasticity, increased abnormal behavior, hyperlocomotion, stereotyped behavior and cognitive defects, all traits associated with ASD (Minichiello et al. 1999; Vyssotski et al. 2002; Zörner et al. 2003).

BDNF is important for spine maturation, axonal and dendritic complexity and synaptic plasticity in specific brain areas and layers (Cohen-Cory and Frazer 1995; McAllister et al. 1995; Lu 2003a, b; Rauskolb et al. 2010) and can cause rapid and long-lasting changes in stimulus-dependent activity in the adult cortex and hippocampus (Kang and Schuman 1995; Prakash et al. 1996; Caleo and Maffei 2002; Lu 2003b). BDNF is secreted in an activity-dependent manner (Egan et al. 2003; Wong et al. 2015). Like all neurotrophins, BDNF can be secreted as a proneurotrophin (pro-BDNF) which may then be cleaved by proteases such as plasmin, prohormone convertases or and matrix metalloproteases to mature BDNF (Lee et al. 2001; Pang et al. 2004; Seidah et al. 1996; Mizoguchi et al. 2011; Wetsel et al. 2013). BDNF and pro-BDNF are both present in the brain (Michalski and Fahnestock 2003), and the proper balance of the two is required for synaptic plasticity and function.

Pro-BDNF and mature BDNF (mBDNF) bind to different receptors to exert opposite biological effects on synapses. Mature BDNF potentiates synaptic proliferation and stabilization, synapse maturation and synaptic transmission through binding to TrkB, and subsequent stimulation of downstream pathways such as PI3 K-Rac, whereas pro-BDNF causes axonal retraction, suppresses synaptic transmission and promotes synapse elimination through binding to p75^NTR^ and stimulation of RhoA (Kaplan and Miller 2000; Roux and Barker 2002; Koshimizu et al. 2009; Yang et al. 2009; Je et al. 2012; Sun et al. 2012). In comparison with controls, individuals with ASD have greater BDNF-like immunoreactivity in the serum, brain and blood compared to controls (Correia et al. 2010; Connolly et al. 2006; Miyazaki et al. 2004; Garcia et al. 2012). Western blotting further revealed that increased BDNF-immunoreactivity in ASD is not due to increased mature BDNF or to changes in BDNF mRNA levels, but that individuals with autism had greater levels of pro-BDNF and less truncated BDNF compared to controls (Garcia et al. 2012). Hence, increased BDNF-immunoreactivity is not transcriptionally driven but may be due to defective conversion of pro-BDNF to the truncated isoform (Fig. 2). The increase in pro-BDNF in idiopathic ASD creates an imbalance between neurotrophic/growth (BDNF/TrkB) and apoptotic/pruning (pro-BDNF/p75^NTR^) (Fahnestock and Nicolini 2015). The altered levels of BDNF isoforms in individuals with ASD might prove to be an integral part of its pathology.

In addition to an imbalance in BDNF isoforms in autism, there is also an imbalance in TrkB isoforms. TrkB is found in full-length (TrkB-FL) and truncated (TrkB-T) isoforms. The truncated isoforms (TrkB-T1 and TrkB-Shc) bind BDNF but lack the catalytic tyrosine kinase domain. Whereas TrkB-FL signals neurotrophic activity through Ras-MAPK, PLC-γ and PI3 K pathways (Kaplan and Miller 2000), the truncated isoforms sequester BDNF, inhibit TrkB-FL signaling and regulate neurite outgrowth via Rho GTPase signaling (Fenner 2012; Wong and Garner 2012). Thus, changes in TrkB isoform balance also lead to alterations in downstream signaling, including the PI3 kinase-dependent pathways Akt-mTOR and Eps8-Rac which govern spine protein synthesis and stability (Fahnestock and Nicolini 2015). This hypothesis is supported by decreased Akt and mTOR total protein and phosphorylation in the cortex of autistic patients (Nicolini et al. 2015; Sheikh et al. 2010) and mutations affecting Akt-mTOR signaling (TSC1/2, PTEN and MeCP2) which cause disorders with high rates of autism (Kelleher and Bear 2008). Mutations in the eIF4E promoter are present in some individuals with autism, and eIF4EBP2 knockout and eIF4E-overexpressing mice (Gkogkas et al. 2013) exhibit autism-like phenotypes, strengthening the hypothesis that disruptions in mTOR-dependent protein synthesis contribute to autism pathology. Lastly, Eps8 protein, which is downstream of BDNF and activates the Rac pathway important for synaptic plasticity, is decreased in idiopathic autism, and knockout of Eps8 in a mouse model resulted in spine abnormalities, decreased LTP and autism-like behavior (Menna et al. 2013). Interestingly, autism-relevant behavior in mouse models can be reversed by administration of mTOR inhibitors (Zhou et al. 2009; Sato et al. 2012) or mTOR pathway activators (Tropea et al. 2009; Schmid et al. 2012). Because BDNF/TrkB-mediated signaling pathways, including Akt-mTOR and Eps8-Rac, play a key role in the development of the cortex and in synaptic function and plasticity (Yoshii and Constantine-Paton 2010), altered BDNF/TrkB signaling through these pathways may be an important contributor to autism pathogenesis.

## Summary of BDNF in Autism

In idiopathic ASD, it has been shown that TrkB-FL levels are reduced, whereas TrkB-T levels are increased (Nicolini et al. 2015). This reduces signaling through PI3 K-mediated pathways (Fig. 2) including Akt-mTOR and Eps8-Rac (Menna et al. 2013; Nicolini et al. 2015). Furthermore, increased pro-BDNF in idiopathic autism (Garcia et al. 2012), acting through p75^NTR^ to activate the Rho pathway, may, together with decreased TrkB signaling through Eps8-Rac, create an imbalance in the Rac/Rho pathways, destabilizing spines and reducing neuritogenesis. Maintaining a balance between the relative levels of BDNF isoforms and their receptors is essential for normal synaptic function and plasticity and for cortical circuitry development. In fact, changes in levels of BDNF and TrkB isoforms are associated with changes in cortical volume in ASD (Raznahan et al. 2009) and deficits in hippocampal function (Egan et al. 2003).

## Effect of Immune Dysregulation on BDNF: Association with TGF-β and Other Tyrosine Kinase Receptors

Neural stem cell maintenance and differentiation are processes that regulate the size of the developing brain. Macrocephaly and increased brain size are a prominent characteristic of ASD neuropathology (Gentile et al. 2013). TGF-β has been identified as a crucial factor regulating these processes. Inflammation can regulate BDNF and other neurotrophin levels within the brain (Guan and Fang 2006). BDNF and TrkB are transcriptionally upregulated by TGF-β (Sometani et al. 2001), whereas the pro-inflammatory cytokine IL-1β reduces BDNF by suppressing its signaling (Tong et al. 2012). Inflammation regulated BDNF signaling may therefore contribute to abnormal neural stem cell development and differentiation by altering the Wnt/β-catenin signaling pathway (Chen et al. 2013). The Wnt signaling pathway and its mediators act along with BDNF to regulate dendritic spine formation and maturation. At least one specific mediator, Wnt2, is regulated by BDNF, and its overexpression leads to increased dendritic growth in cortical neurons (Hiester et al. 2013; Yi et al. 2012).

TGF-β/Tgfbr2 (TGF-β receptor) gene mediates these effects through regulated expression of Wnt-1/β-catenin and FGF8 target genes (Fig. 2). TGF-β signaling counteracts Wnt-mediated proliferation of the neuroepithelium and thus regulates brain size and regionalization (Falk et al. 2008). The WNT signaling pathway is expressed under strict regulatory and spatially specific control during forebrain development and hence plays an integral role in establishing regional forebrain identity. Studies have now shown that both hypo- and hyper-activity of the WNT pathway are associated with ASD phenotypes (Kalkman 2012). In support of this, the WNT2 gene, besides being located in a chromosomal region increasingly associated with autism, i.e., 7q31, is also associated with ASD in humans (Marui et al. 2010).

Neuroepithelial expansion and cell-type specification is regulated by WNT signaling in the cortex. WNT, along with Bmp, FGF and Shh signaling pathways, also controls dorsoventral patterning of the forebrain (Kwan et al. 2016; Fig. 2). For example, loss of FGF17 reduces the size of the dorsal frontal cortex and leads to autistic behaviors (Cholfin and Rubenstein 2007). Loss of FGF17 has also been shown to be responsible for impaired cognitive and social behavior with the inability to respond to social information in a mouse knockout model (Scearce-Levie et al. 2008). Thus, functional crosstalk between TGF-β and Wnt pathways and synaptogenic growth factors such as BDNF and FGF in specific brain regions could have implications for the development of the human brain (Fig. 2) and its disorders (Falk et al. 2008). Other ASD-associated signaling proteins could also interact with these pathways and influence pathophysiology. For example, mutants of TGFβ receptor binding partners, such as the semaphorin 3F-neuropilin 2 system, display seizure activity, epileptiform EEGs, interneuron reductions, altered synaptogenesis and synaptic pruning, impaired synaptic plasticity and autistic like behaviors (Gant et al. 2009). Thus, this crosstalk of signaling processes alters terminal differentiation in the later stages of development by promoting adaptive or mal-adaptive neurogenesis (Harrison-Uy et al. 2012).

## Implications of the Immune-Synaptogenic Growth Factor Hypothesis

**Fig. 3:**
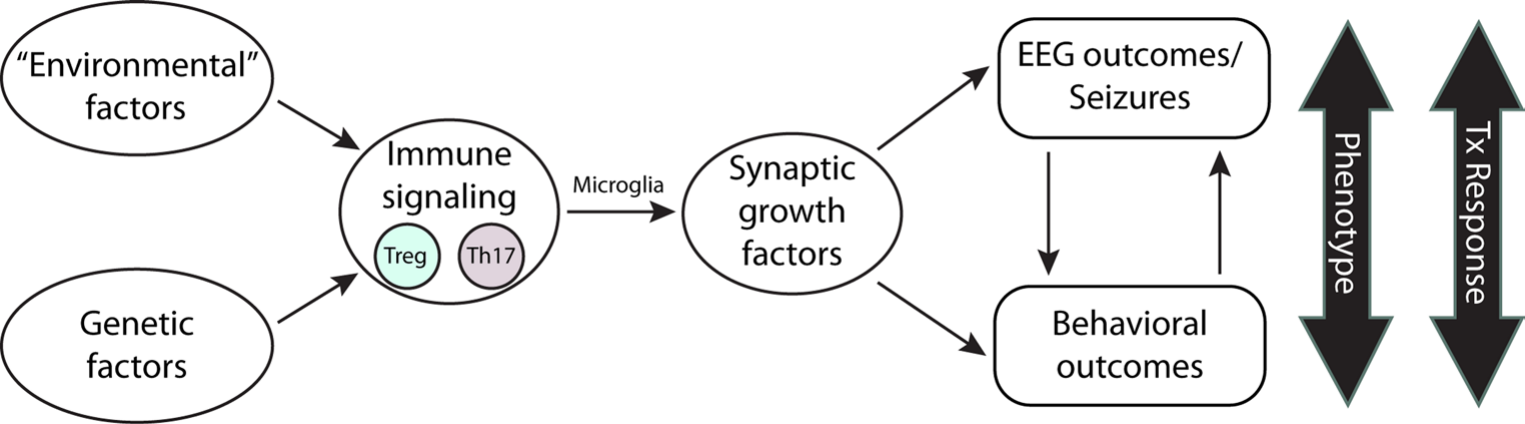
Environmental and genetic influences of ASD phenotypes and treatment responses. Environmental and genetic factors may cause immune dysregulation via altered T cell subtype production (Th1/Th2/Th17/Treg), thereby influencing microglial functioning. The impact of altered microglial cell physiology leads to dysregulation of synaptic growth factors and signaling pathways (BDNF/WNT, FGF and their intracellular components–PI3K/Akt/mTOR signaling) which contributes to ASD phenotypes and treatment responses. Tx = treatment

In addition to their role in CNS inflammation, microglia have been implicated in normal neurogenesis and formation of synapses and neural networks during CNS development (Nimmerjanhn et al. 2005; Parkhurst et al. 2013). Additionally, microglia play a role in normal post-natal apoptosis pruning neuronal connections and controlling the number of synapses and their maturation (Shatz 1990; Paolicelli et al. 2011; Schafer et al. 2013). Complement activation has also been implicated in pruning. Disruption of CR3/C3 signaling leads to increased synapse numbers and connectivity (Schafer et al. 2012; Stephan et al. 2012). Mice deficient in the fractalkine receptor CX3CR1, specifically expressed on microglia, had decreased functional brain connectivity, reduced synaptic pruning, abnormal electrophysiological responses and displayed impaired social interaction and increased repetitive (ASD-like) behavior, further implicating microglial signaling in ASD mechanisms (Zhan et al. 2014). The increased brain microglial density and activated morphology may be the result of altered cytokine signaling and Th17/Treg cell physiology in the brain (Fig. 3; Morgan et al. 2010; Edmonson et al. 2014). The decrease in serum TGF-β and increased microglial production of TGF-β within the brain parenchyma of those with ASD may alter the processing of the BDNF/TrkB molecules in ASD.

In addition to the genetic etiology of ASD, environmental factors such as maternal infection, obesity, metal levels and inflammatory responses may contribute to the disease development (Tanako 2015). Thus, disruption of normal microglial biology by immune dysregulation and altered neurotrophin signaling likely contributes to the pathogenesis of ASD, including epileptiform EEGs, seizures and poor behavioral outcomes (Fig. 3). Further investigations into these factors and identification of small molecule therapeutics will likely yield better treatment responses with ASD immune subgroup(s).

## Suggestions for Further Research

Further research is needed to assess immune system functioning in ASD. Analysis of Th17 levels and Treg cell functioning and levels of associated cytokines (IL-17, TGF-β, IL-21, IL-23, IL-6, IL-4, IL-1beta) may help to identify intra- and inter-group differences between children with ASD and age-matched controls and to stratify ASD subtypes based on hyper- or hypo-functioning of the immune system. In particular, ASD phenotypes with inflammatory components such as GI disturbances should be considered in such analyses. Further research is essential to establish a firm relationship between TFG-β and the WNT signaling pathway, and exome sequencing is required to assess genotype–phenotype differences within and between gene clusters involving the WNT, TGF-β and interleukin pathways. Analysis of functional and developmental consequences of alterations in synaptogenic growth factors (FGF and BDNF family of proteins) in children and adolescents with ASD and their interaction with the TGF-β/Wnt signaling pathway is also needed. Levels of FGF and BDNF family of proteins and their isoforms may influence the severity or type of symptoms in children and adolescents with ASD compared to their age-matched controls (Fig. 3). Establishing the relationship between TFG-β signaling, BDNF/FGF signaling and measured indicators of WNT signaling pathways is needed.

## Conclusions

While tremendous progress has been made in the field, ASD continues to be an elusive disorder. Numerous theories have been developed, but there remains a lack of specific markers, immune, neurochemical or genetic, that aid in establishing the pathogenesis and subgrouping of ASD. We attempt to unravel connections between existing markers via their normal physiological roles and offer an explanation for a pathogenic course that might exist in a group of children with ASD. This may afford an opportunity to adopt a personalized approach to treating these children accordingly.
